# Interactive Natural Language Grounding via Referring Expression Comprehension and Scene Graph Parsing

**DOI:** 10.3389/fnbot.2020.00043

**Published:** 2020-06-25

**Authors:** Jinpeng Mi, Jianzhi Lyu, Song Tang, Qingdu Li, Jianwei Zhang

**Affiliations:** ^1^Institute of Machine Intelligence (IMI), University of Shanghai for Science and Technology, Shanghai, China; ^2^Technical Aspects of Multimodal Systems, Department of Informatics, University of Hamburg, Hamburg, Germany

**Keywords:** interactive natural language grounding, referring expression comprehension, scene graph, visual and textual semantics, human-robot interaction

## Abstract

Natural language provides an intuitive and effective interaction interface between human beings and robots. Currently, multiple approaches are presented to address natural language visual grounding for human-robot interaction. However, most of the existing approaches handle the ambiguity of natural language queries and achieve target objects grounding via dialogue systems, which make the interactions cumbersome and time-consuming. In contrast, we address interactive natural language grounding without auxiliary information. Specifically, we first propose a referring expression comprehension network to ground natural referring expressions. The referring expression comprehension network excavates the visual semantics via a visual semantic-aware network, and exploits the rich linguistic contexts in expressions by a language attention network. Furthermore, we combine the referring expression comprehension network with scene graph parsing to achieve unrestricted and complicated natural language grounding. Finally, we validate the performance of the referring expression comprehension network on three public datasets, and we also evaluate the effectiveness of the interactive natural language grounding architecture by conducting extensive natural language query groundings in different household scenarios.

## 1. Introduction

Natural language grounding aims to locate target objects within images given natural language queries, and grounding natural language queries in visual scenes can create a natural communication channel between human beings, physical environments, and intelligent agents. Moreover, natural language grounding is widely used in image retrieval (Gordo et al., 2016), visual question answering (Li et al., 2018), and robotics (Paul et al., 2018; Mi et al., 2019).

With applications of robots becoming omnipresent in varied human environments such as factories, hospitals, and homes, the demand for natural and effective human-robot interaction (HRI) has become urgent. Natural language grounding-based HRI is also attracting considerable attention, and multiple approaches have been proposed (Schiffer et al., 2012; Steels et al., 2012; Twiefel et al., 2016; Ahn et al., 2018; Hatori et al., 2018; Paul et al., 2018; Shridhar and Hsu, 2018; Mi et al., 2019; Patki et al., 2019).

Natural language grounding-based HRI requires a comprehensive understanding of natural language instructions and working scenarios, and the pivotal issue of is to locate the referred objects in working scenarios according to given instructions. Although the existing models achieve promising results, some of them either do not take the inherent ambiguity of natural language into consideration (Paul et al., 2018; Katsumata et al., 2019; Mi et al., 2019; Patki et al., 2019), or alleviate the ambiguity via drawing support from auxiliary information, such as dialogue system (Ahn et al., 2018; Hatori et al., 2018; Shridhar and Hsu, 2018) and gestures (Shridhar and Hsu, 2018). However, the dialogue-based disambiguation systems entail time cost and cumbersome interactions.

Tasks that utilize textual descriptions or questions to help human beings to understand or depict images and scenes are in agreement with the human desire to understand visual contents at a high semantic level. Examples of these tasks include dense captioning (Johnson et al., 2016), visual question answering (Antol et al., 2015), referring expression comprehension (Yu et al., 2016), etc. Referring expression comprehension imitates the role of a listener to locate target objects within images given referring expressions. Compared to other tasks, referring expression comprehension focuses on objects in visual images and locates specific targets via modeling the relationship between objects and referring expressions.

Inspired by the role of referring expression comprehension, we propose an interactive natural language grounding architecture based on referring expression comprehension. Specifically, we propose a semantic-aware network for referring expression comprehension task. The proposed semantic-aware network is composed of a visual semantic-aware network, a language attention network, and a target localization module. The visual semantic-aware network highlights the visual semantics of regions by fully utilizing the characteristics of deep features extracted from a pretrained CNN (Convolutional Neural Network). The language attention network learns to assign different weights to each word in expressions and parse expressions into phrases that embed information of target candidate, relation between objects, and spatial location, respectively. And the target localization module combines the visual and textual representations to locate target objects. We train the proposed network on three popular referring expression datasets: RefCOCO (Yu et al., 2016), RefCOCO+ (Yu et al., 2016), and RefCOCOg (Mao et al., 2016).

In real applications, natural language queries are complicated and ambiguous. While the expressions in the referring expression datasets are simple sentences and only indicate one target, so the complicated queries can not be grounded only by the trained referring expression comprehension model. Inspired by the role of scene graph which describes objects within visual images and the relationship between objects, we integrate the referring expression comprehension network with scene graph parsing (Johnson et al., 2015) to ground unconstrained and complicated natural language queries.

Moreover, we conduct extensive experiments on test sets of the three referring expression datasets to validate the proposed referring expression comprehension network. In order to evaluate the performance of the interactive natural language grounding architecture, we collect plenty of indoor working scenarios and diverse natural language queries. Experimental results demonstrate that the presented natural language grounding architecture can ground complicated queries without the support from auxiliary information.

To sum up, our major contributions are two-fold. First, we propose a semantic-aware network for referring expression comprehension, in which we take full advantage of the characteristics of the deep features and exploit the rich contexts of referring expressions. Second, we present a novel interactive natural language grounding architecture by combining the referring expression comprehension network with scene graph parsing to ground complicated natural language queries.

## 2. Related Work

### 2.1. Natural Language Grounding for HRI

Multiple approaches have been proposed to address natural language grounding for HRI. Schiffer et al. (2012) adopted decision-theoretic planning to interpret spoken language commands for natural language-based HRI in domestic service robotic applications. Steels et al. (2012) presented Fluid Construction Grammar (FCG) to understand natural language sentences, and FCG was suitable for real robot requires because of its robustness and flexibility. Fasola and Matarić (2014) proposed a probabilistic method for service robots to interpret spatial language instructions.

Twiefel et al. (2016) combined an object classification network, a language understanding module with a knowledge base to understand spoken commands. Paul et al. (2018) proposed a probabilistic model named adaptive distributed correspondence graph to understand abstract spatial concepts, and an approximate inference procedure to realize concrete constituents grounding. Patki et al. (2019) utilized distributed correspondence graph to infer the environment representation in a task-specific approach. Katsumata et al. (2019) introduced a statistical semantic mapping method that enables the robot to connect multiple words embedded in spoken utterance to a place in a semantic mapping processing. However, these models did not take into account the inherent vagueness of natural language. Our previous work (Mi et al., 2019) first presented an object affordances detection model, and then integrated the object affordances detection with a semantic extraction module for grounding intention-related spoken language instructions. This model, however, was subject to limited categories of affordances, so it can not ground unconstrained natural language.

Shridhar and Hsu (2018) adopted a pretrained captioning model, DenseCap (Johnson et al., 2016), to generate expressions for detected regions in uncluttered working scenarios, and through conducting K-means clustering to identify the relativeness of input instructions and the generated expressions. The expressions generated by DenseCap (Johnson et al., 2016) do not include the interaction information between objects, such as the relationship between objects. Therefore, the authors of work (Shridhar and Hsu, 2018) employed gestures and a dialogue system to disambiguate spoken instructions. Hatori et al. (2018) drew support from a referring expression comprehension model (Yu et al., 2017) to identify the target candidates, and tackled with the ambiguity of spoken instructions via conversation between human users and robots. Ahn et al. (2018) first employed hourglass network (Newell et al., 2016) to generate position heatmap for working scenarios, and combined the generated heatmap with a question generation module to locate targets according to the answers for the generated questions. Thomason et al. (2019) translated the spoken instructions into discrete robot actions and improved objects grounding through clarification conversations with human users. Nevertheless, dialogue systems usually make HRI cumbersome and time-consuming.

Thomason et al. (2016) took into account visual, haptic, auditory, and proprioceptive data to predict the target objects, and the natural language grounding supervised by an interactive game. However, this model needs to gather language labels for objects to learn lexical semantics. Magassouba et al. (2018) presented a multi-modal classifier generative adversarial network to enable robots to implement carry-and-place tasks, and disambiguates the natural language commands by utilizing the contexts of working environments and the states of the robots.

By contrast, we disambiguate natural language queries by a referring expression comprehension network and achieve interactive natural language grounding without auxiliary information. To alleviate the ambiguity of natural language queries, we take into consideration the relations, the region visual appearance difference, and the spatial location information during the referring expression comprehension network training. Besides, we integrate the trained referring expression comprehension model with scene graph parsing to achieve unrestricted and complicated interactive natural language grounding.

### 2.2. Referring Expression Comprehension

Referring expression comprehension aims to locate the most related objects in images according to given referring expressions. Compared with image captioning and visual question answering, referring expression comprehension is widely used in image retrieval (Chen k. et al., 2017), video question answering (Gao et al., 2017), and natural language based HRI (Hatori et al., 2018; Shridhar and Hsu, 2018).

In terms of representations of image regions and natural language referring expressions, existing approaches for referring expression comprehension can be generalized into two categories: (1) visual representations un-enriched models, which directly extract deep features from a pretrained CNN as the visual representations of detected image regions (Mao et al., 2016; Yu et al., 2016, 2017; Hu et al., 2017; Deng et al., 2018; Zhang et al., 2018; Zhuang et al., 2018). (2) visual representations enriched models, which enhance the visual representations by adding external visual information for regions (Liu et al., 2017; Yu et al., 2018a,b). Liu et al. (2017) leveraged external knowledge acquired by an attributes learning model to enrich the information of regions. Yu et al. (2018b) trained an object detector on the Visual Genome dataset (Krishna et al., 2017) to generate diversified and discriminative proposals. Yu et al. (2018a) extracted deep features from two different convolutional layers to predict region attribute cues. However, these mentioned approaches neglected the rich information embedded in the extracted deep features.

Attention mechanism was introduced for image captioning (Xu et al., 2015) and become an indispensable component in deep models to acquire superior results (Anderson et al., 2018; Yu et al., 2018a). Due to the excellent performance of attention mechanisms, they have also been utilized in referring expression comprehension (Hu et al., 2017; Deng et al., 2018; Yu et al., 2018a; Zhuang et al., 2018). Hu et al. (2017) parsed the referring expressions into a triplet (subject, relationship, object) by an external language parser, and computes the weight of each part of parsed expressions with soft attention. Deng et al. (2018) introduced an accumulated attention network that accumulated the attention information in image, objects, and referring expression to infer targets. Zhuang et al. (2018) argued that the image representation should be region-wise, and adopted a parallel attention network to ground target objects recurrently. Notwithstanding, these models processed expressions as holistic and ignored the rich context of expressions. Wang et al. (2019) introduced a graph-based attention mechanism to address the target candidates and the relationships between objects within images, while the visual semantic in images was neglected.

Unlike the above mentioned approaches, we address the visual semantics of regions by taking advantage of the inherent semantic attributes of deep features, i.e., channel-wise and spatial characteristics of extracted deep features. Additionally, we explore the textual semantics by adopting BERT to generate word representations and employ a language attention network to learn to decompose expressions into phrases to ground target objects.

## 3. Architecture Overview

**Figure 1:**
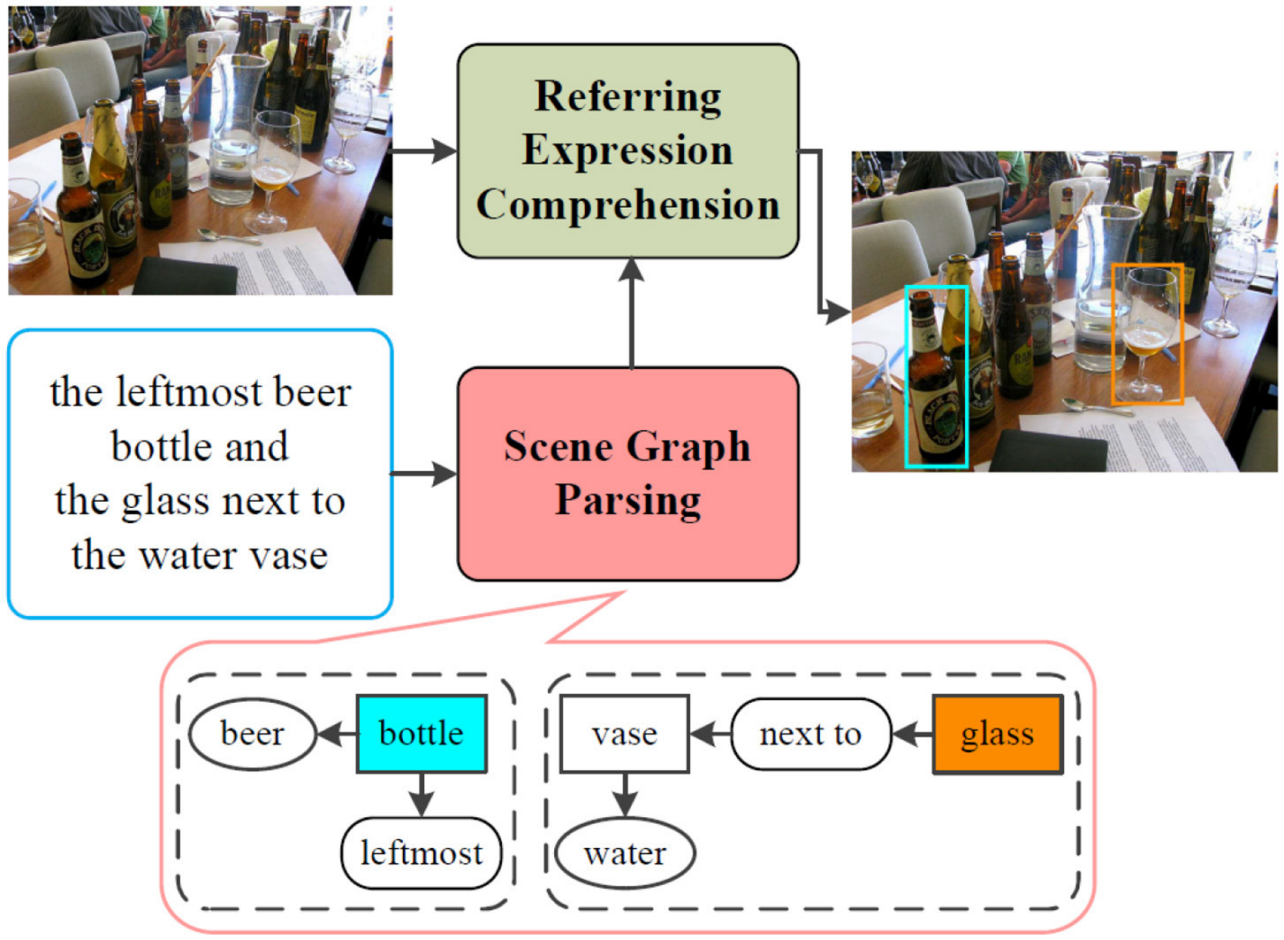
The architectural diagram of the proposed interactive natural language grounding. We first parse the interactive natural language queries into scene graph legends by the scene graph parsing. We then ground the generated scene graph legends via the referring expression comprehension network. The mark rectangle in bottom encompasses the scene graph parsing result for the input natural language query. The rounded rectangles with black dashed lines denote the parsed scene graph legends, color shaded rectangles represent referents, no color shaded rectangle is an object, ovals indicate objects attributes, rounded rectangles act for edges which indicate relations between target and other objects. The same color of the bounding boxes in the output image and the referents in the generated scene graph legends denotes a grounding.

Natural language provides a more intuitive interface to achieve natural and effective HRI. For grounding unrestricted and complicated interactive natural language queries, we propose a novel architecture, as shown in Figure 1. We decompose the interactive natural language grounding into two subtasks: (1) parse the complicated natural language queries into scene graph legends by scene graph parsing. The scene graph legend is a data structure consisting of nodes that denote objects with attributes and edges that indicate the relations between objects; (2) ground the parsed natural language queries by the referring expression comprehension network.

In this work, we aim to locate the most related referents in working scenarios given interactive natural language expressions without auxiliary information. The inputs consist of a working scenario given as an RGB image and an interactive natural language instruction given as text, and the outputs are the bounding boxes of target objects. We parse the input natural language instructions into scene graph legends by scene graph parsing, and then we ground the acquired scene graph legends via the referring expression comprehension network.

We elaborate the details of the referring expression comprehension network in section 4, and we describe the scene graph parsing in section 5. Following this, we outline the experiments conducted to evaluate the referring expression comprehension network and the interactive natural language grounding architecture in section 6.

## 4. Referring Expression Comprehension via Semantic-Aware Network

**Figure 2:**
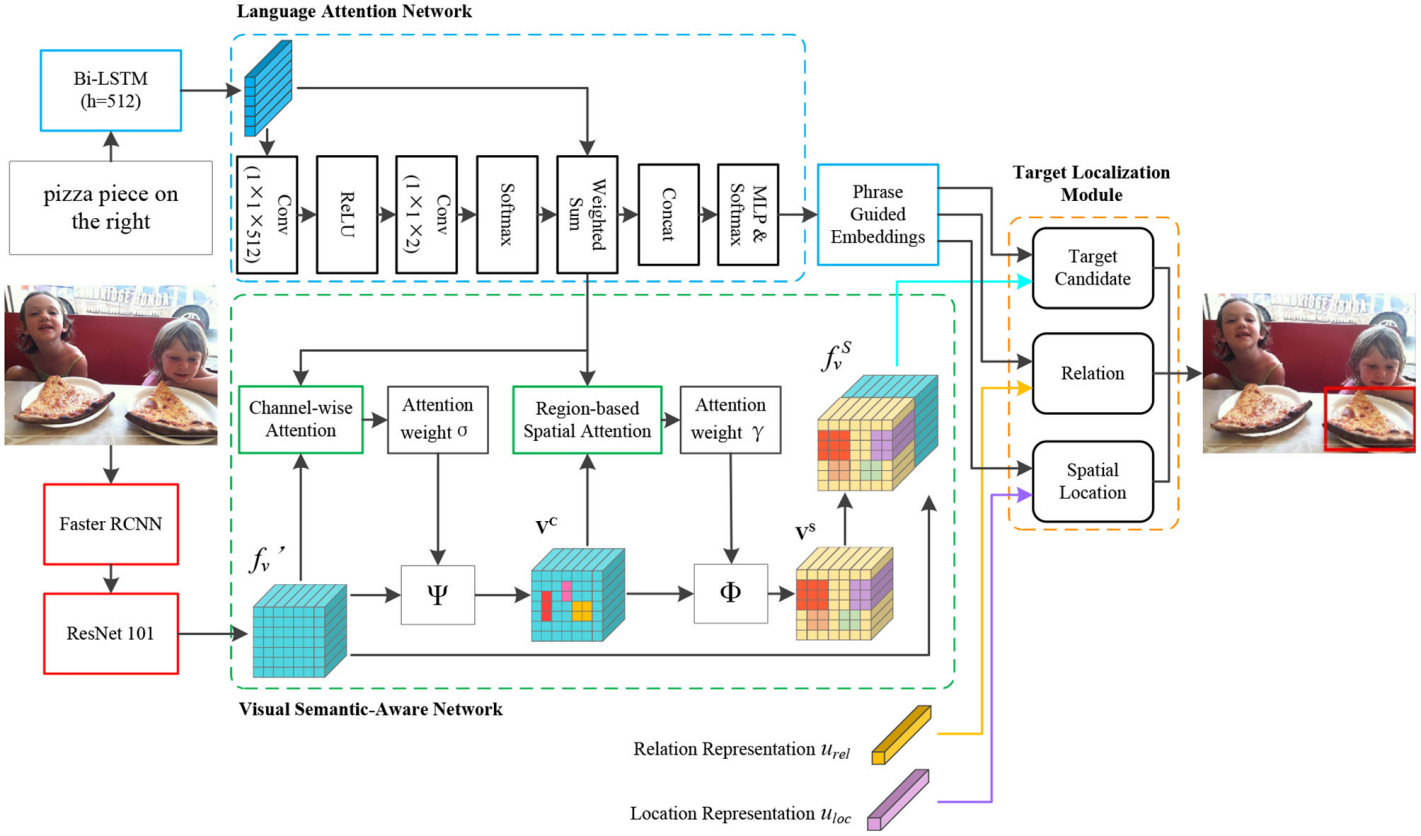
Semantic-Aware network for referring expression comprehension. We adopt the language attention network to compute the different weights for each word in expressions, and learn to parse the expressions into phrases that embed the information of target candidate, relation, and spatial location, respectively. We conduct both channel-wise and region-based spatial attention to generate semantic-aware region visual representation. We further combine the outputs of the visual semantic-aware network, the language attention network, and the relation and location representations to locate the target objects. In the figure, $f_{v}^{\prime }$ denotes the projected deep features, **V**^*C*^ represents the channel-wise weighted deep feature, **V**^*S*^ is the spatial weighted feature, $f_{v}^{S}$ is the generated semantic-aware visual representation by concatenating $f_{v}^{\prime }$ and **V**^*S*^, the details are described in section 4.2. The relation representation *u*_*rel*_, the location representation *u*_*loc*_, and the details of the target candidate module, the relation module, and the location module are introduced in section 4.3. Ψ denotes a channel-wise multiplication for $f_{v}^{\prime }$ and the generated channel-wise attention weight σ, Φ represents element-wise multiplication for **V**^*C*^ and the acquired spatial attention weight γ (Best viewed in color).

Given a referring expression *r* with *M* words *r* = ${\left\{w_{i}\right\}}_{i=1}^{M}$ and an image *I* with *N* region of interests (RoIs) *I* = ${\left\{o_{j}\right\}}_{j=1}^{N}$, we model the relation between *w*_*i*_ and *o*_*j*_ to locate the target object. In this study, we propose a referring expression comprehension network comprises: (1) a language attention network learns to assign different weights to each word in expressions, and parse the expressions into phrases that denote target candidate, relation between target candidate and other objects, and location information; (2) a visual semantic-aware network generates semantic-aware visual representation, which is acquired by the channel-wise and the region-based spatial attention; (3) a target localization module achieves targets grounding by combining the outputs of the language attention network, the output of the visual semantic-aware network with the components of the target localization module. Figure 2 illustrates the details of the proposed semantic-aware network for referring expression comprehension.

### 4.1. Language Attention Network

We propose a language attention network to learn the different weights of each word in referring expressions, and also to learn to parse the expressions into target candidate embedding *r*_*tar*_, relation embedding *r*_*rel*_, and spatial location embedding *r*_*loc*_, respectively.

For an expression *r*, we employ BERT (Devlin et al., 2019) to tokenize and encode *r* into contextualized word embeddings *E*_*r*_ = [*e*_1_, *e*_2_,., *e*_*M*_], where *e*_*i*_ ∈ ℝ^1 × 1024^. We then feed *E*_*r*_ into an one-layer BiLSTM:

(1) $$\begin{array}{l} L_{out}=\text{BiLSTM}\left(E_{r}\right) \end{array}$$

where *L*_*out*_ is the output of the BiLSTM.

To acquire the different weight of each word, we compute attention distribution over the expressions by:

(2) $$\begin{array}{l} \alpha_{l}=\text{softmax}(F(L_{out}))\\ L=\sum_{i}^{g}\alpha_{l,i}L_{out,i} \end{array}$$

where α_*l*_ denotes the calculated attention weight, and $\overset{M}{\underset{m=1}{\sum }}\alpha_{l}$ = 1. In the implementation, F is modeled by two convolution layers. The generated expression representation *L* ∈ ℝ^d × 2048^, *d* is length of expressions in different dataset.

Expressions like “cup with printed red flowers,” some words should be parsed to a phrase to represent specific information, e.g., “with printed red flowers.” To this end, we employ a single perceptron layer and a softmax layer to learn to parse the expression into three module embeddings:

(3) $$\begin{array}{l} \bar{L}=\varphi \left(W_{t}L+b_{t}\right)\\ \left[w_{tar},w_{loc},w_{rel}\right]=\text{softmax}\left(\bar{L}\right) \end{array}$$

where φ is a non-linear activation function, in this paper, we use hyperbolic tangent. *W*_*t*_ is a trainable weight matrix and *b*_*t*_ represents a bias vector. *w*_*tar*_, *w*_*loc*_, *w*_*rel*_ represent weights guided by target embedding *r*_*tar*_, relation embedding *r*_*rel*_, and spatial location embedding *r*_*loc*_, respectively.

### 4.2. Visual Semantic-Aware Network

We take full advantage of the characteristics of deep features extracted from a pretrained CNN model, and we conduct channel-wise and region-based spatial attention to generate semantic-aware visual representation for each detected region. This process can be deemed as visual representation enrichment for the detected regions.

#### 4.2.1. RoI Features

Given an image, we adopt Faster R-CNN (Ren et al., 2015) to generate RoIs, and we extract deep feature *f*_*v*_ ∈ ℝ^7 × 7 × 2048^ for each *o*_*j*_ from the last convolutional layer of the 4th-stage of ResNet101 (He et al., 2016), where 7 × 7 denotes the size of the extracted deep feature, 2048 is the output dimension of the convolutional layer, i.e., the number of channels. We then project the deep feature *f*_*v*_ into a 512-dimension subspace by a convolution operator with 1 × 1 kernel, i.e., the projected deep feature $f_{v}^{\prime }$ ∈ ℝ^7 × 7 × 512^.

#### 4.2.2. Channel-Wise Attention

Essentially, deep features extracted from CNN are spatial, channel-wise, and multi-layer. Each channel of a deep feature correlates with a convolutional filter which performs as a pattern detector (Chen L. et al., 2017). For example, the filters in lower layers detect visual clues such as color and edge, while the filters in higher layers capture abstract content such as object component or semantic attributes. Accordingly, performing channel-wise attention on higher-layer features can be deemed as a process of semantic attributes selection.

We first reshape the projected RoI deep feature *f_v_^′^* to **V**=[**v**_**1**_, **v**_**2**_, ., **v**_***d*_*v*_**_], where **v**_***i***_ ∈ ℝ^7 × 7^ is the *i*-th channel of the deep feature *f_v_^′^*, *d*_*v*_=512. We then perform average pooling on each channel to generate the channel-wise vector *V* = [*v*_1_, *v*_2_, ..., *v*_*d*_*v*__], where *V* ∈ ℝ^1 × 512^, *v*_*i*_ represents the *i*-th pooled channel feature.

After the feature pooling, we first utilize L2-normalization to process channel-wise vector *V* and expression representation *L* to generate more robust representations, we then perform channel-wise attention by a channel-wise attention network which is composed of an MLP (multi-layer perceptron) and a softmax layer. For the detected image region, the input of the attention network is average-pooled feature *V* and the weighted expression representation *L*. The channel-wise attention weight is acquired by:

(4) $$\begin{array}{l} A_{c}=\varphi \left(\left(W_{v,c}V+b_{v,c}\right)⊗\left(W_{t,c}L+b_{t,c}\right)\right)\\ \sigma =\text{softmax}\left(A_{c}\right) \end{array}$$

where *W*_*v, c*_ and *W*_*t, c*_ are learnable weight matrices, *b*_*v, c*_ and *b*_*t, c*_ are bias vectors, *W*_*v, c*_ and *b*_*v, c*_ are the parameters of the MLP for visual representation, while *W*_*t, c*_ and *b*_*t, c*_ for textual representation. ⊗ denotes outer product, σ ∈ ℝ^1 × 512^ is the learned channel-wise attention weight which encodes the semantic attributes of regions. In the following, *W*_*v*, ._ and *b*_*v*, ._ represent the weight matrix and bias vector for visual representation, while *W*_*t*, ._ and *b*_*t*, ._ denote the trainable parameters for textual representation.

#### 4.2.3. Region-Based Spatial Attention

The channel-wise attention attempts to address the semantic attributes of regions, while the region-based spatial attention is employed to attach more importance to the referring expressions related regions. To acquire region-based spatial attention weights, we first combine the learned channel-wise attention weight σ with the projected deep feature $f_{v}^{\prime }$ to generate channel-wise weighted deep feature **V**^***C***^.

(5) $${\text{V}}^{C}=\Psi (f_{v}\prime ,\sigma )$$

where Ψ is a channel-wise multiplication for deep feature channel and the corresponding channel weights, **V**^***C***^ ∈ ℝ^49 × 512^.

We put the weighted channel-wise deep features **V**^***C***^ and the weighted expressions into an attention network similar to the channel-wise attention to calculate the spatial attention γ:

(6) $$\begin{array}{l} A_{s}=\varphi \left(\left(W_{v,s}{\text{V}}^{C}+b_{v,s}\right)⊗\left(W_{t,s}L+b_{t,s}\right)\right)\\ \gamma =\text{softmax}\left(A_{s}\right) \end{array}$$

The acquired γ ∈ ℝ^49 × 1^ denotes the weight of each region related to the expressions. We further fuse the γ with channel-wise weighted feature **V**^***C***^ to obtain spatial weighted deep feature **V**^***S***^:

(7) $$\begin{array}{l} {\text{V}}^{S}=\Phi \left({\text{V}}^{C},\gamma \right) \end{array}$$

where Φ denotes element-wise multiplication for generated **V**^***C***^ and the corresponding γ.

Spatial weighted deep feature **V**^***S***^ ∈ ℝ^7 × 7 × 512^ comprises the semantics guided by the channel-wise attention as well as the spatial weight of each region. Therefore, we define **V**^***S***^ as semantic-aware deep feature. Finally, we concatenate **V**^***S***^ with projected feature $f_{v}^{\prime }$ to obtain semantic-aware visual representation for each region, i.e., $f_{v}^{S}$ = [$f_{v}^{\prime }$ ; **V**^***S***^], $f_{v}^{S}$ ∈ ℝ^7 × 7 × 1024^, [· ; ·] denotes the concatenate operation.

### 4.3. Target Localization Module

In order to locate target objects for given expressions, we need to sort out the relevant candidates, the spatial location, and the appearance difference between the candidate and other objects. For instance, to understand the expression “the cow directly to the right of the largest cow,” we need to understand the spatial location “the right of,” and the appearance difference “largest” between the cows to identify the target “cow.” To this end, we deal with the relevant candidates, the relation and spatial location through a target candidate module, a relation module, and a spatial location module, respectively.

#### 4.3.1. Target Candidate Module

We compute the target candidate phrase matching score by the target candidate module. For a given region semantic-aware representation $f_{v}^{S}$ and target candidate phrase guided expression embedding *r*_*tar*_, we process $f_{v}^{S}$ and *r*_*tar*_ by L2-normalization and linear transform to compute the attention weights on each region:

(8) $$\begin{array}{l} t=\varphi \left(\left(W_{v}f_{v}^{S}+b_{v}\right)⊗\left(W_{t}r_{tar}+b_{t}\right)\right)\\ \beta =\text{softmax}\left(t\right) \end{array}$$

where β denotes the learned region-based attention weight.

We fuse β and $f_{v}^{S}$ to obtain the target candidate phrase attended region visual representation *u*_*tar*_, and we further compute the target candidate matching score *s*_*tar*_ by:

(9) $$\begin{array}{l} u_{tar}=\beta ⊗f_{v}^{S}\\ {\bar{u}}_{tar}=W_{v,tar}u_{tar}+b_{v,tar}\\ {\bar{r}}_{tar}=W_{t,tar}r_{tar}+b_{t,tar}\\ s_{tar}=D\left({\bar{u}}_{tar},{\bar{r}}_{tar}\right) \end{array}$$

where D(·, ·) represents the consine distance measurement.

#### 4.3.2. Relation Module

We adopt a relation module to obtain the matching score of a pair of candidates and relation embedding *r*_*rel*_. We use the average-pooled channel vector *V* as the appearance representation for each candidate. To tackle with the appearance difference between candidates, e.g., “the largest cow,” we calculate the visual appearance difference representation δ*v*_*i*_=$\frac{1}{n}$$\underset{j\neq i}{\sum }\frac{v_{i}-v_{j}}{\left||v_{i}-v_{j}|\right|}$ as (Yu et al., 2016), where *n* is the number of candidates chosen for caparison (in our implementation *n* = 5). We concatenate *V* and δ*v*_*i*_ as the candidates visual relation representation *u*_*rel*_, i.e., *u*_*rel*_ = [*V* ; δ*v*_*i*_]. We calculate the relation matching score by:

(10) $$\begin{array}{l} {\bar{u}}_{rel}=W_{v,rel}u_{rel}+b_{v,rel}\\ {\bar{r}}_{rel}=W_{t,rel}r_{rel}+b_{t,rel}\\ s_{rel}=D\left({\bar{u}}_{rel},{\bar{r}}_{rel}\right) \end{array}$$

#### 4.3.3. Spatial Location Module

We calculate the location matching score through the location module. To deal with the spatial relation of candidates in images, following (Yu et al., 2016), we adopt a 5-dimensional spatial vector *u*_*l*_ = [$\frac{x_{tl}}{W}$, $\frac{y_{tl}}{H}$, $\frac{x_{br}}{W}$, $\frac{y_{br}}{H}$, $\frac{w\cdot h}{W\cdot H}$] to encode the top left position, bottom right position, and the relative size of the candidates in images. In order to address the relative position expression like “the right of,” “in the middle,” we adopt the relative location vector Δ*u*_*ij*_ = [$\frac{{\left[\Delta x_{tl}\right]}_{ij}}{w_{i}}$, $\frac{{\left[\Delta y_{tl}\right]}_{ij}}{h_{i}}$, $\frac{{\left[\Delta x_{br}\right]}_{ij}}{w_{i}}$, $\frac{{\left[\Delta y_{br}\right]}_{ij}}{h_{i}}$, $\frac{w_{j}\cdot h_{j}}{w_{i}\cdot h_{i}}$] which is obtained by comparing with five surrounding objects and concatenate with *u*_*l*_ to generate candidate location representation *u*_*loc*_ = [*u*_*l*_ ; Δ*u*_*ij*_].

Similar to the target candidate module, we process *u*_*loc*_ and location phrase *r*_*loc*_, and then combine the transformed *u*_*loc*_ and *r*_*loc*_ to generate the location matching score *s*_*loc*_:

(11) $$\begin{array}{l} {\bar{u}}_{loc}=W_{v,loc}u_{loc}+b_{v,loc}\\ {\bar{r}}_{loc}=W_{t,loc}r_{loc}+b_{t,loc}\\ s_{loc}=D\left({\bar{u}}_{loc},{\bar{r}}_{loc}\right) \end{array}$$

### 4.4. Learning Objective

Given a referring expression *r* and an image *I* with multiple RoIs pair, we calculate the target candidate score, the relation score, and the location score, through the three above introduced modules. We locate the target object by the final grounding score:

(12) $$\begin{array}{l} G\left(o_{i}|r\right)=w_{tar}s_{tar}+w_{rel}s_{rel}+w_{loc}s_{loc} \end{array}$$

In the implementation, we adopt a combined max-margin loss as the objective function:

(13) $$\begin{array}{l} L_{\theta }=\sum_{i}\left[max(0,\xi -G(o_{i}|r_{i})+G(o_{i}|r_{j})\right)\\ \;+max(0,\xi -G(o_{i}|r_{i})+G(o_{k}|r_{i}))] \end{array}$$

where θ denotes the parameters of the model to be optimized, ξ is the margin between positive and negative samples. During training, we set ξ = 0.1. For each positive target and expression pair (*o*_*i*_, *r*_*i*_), we randomly select negative pairs (*o*_*i*_, *r*_*j*_) and (*o*_*k*_, *r*_*i*_), where *r*_*j*_ is the expression for other objects, *o*_*k*_ is the other object in the same image.

## 5. Scene Graph Parsing

The introduced referring expression comprehension network is trained on RefCOCO, RefCOCO+, and RefCOCOg. The referring expressions in RefCOCO and RefCOCO+ were collected by an interactive manner (Kazemzadeh et al., 2014), and the average length of expressions in RefCOCO is 3.61, and the average number of words in RefCOCO+ expressions is 3.53. While RefCOCOg expressions were collected in a non-interactive way, therefore produces longer expressions and the average length is 8.43. From the perspective of expression length distribution, 97.16% expressions in RefCOCO contain less than 9 words, the proportion in RefCOCO+ is 97.06%, while 56.0% expressions in RefCOCOg comprise less than 9 words. Moreover, the expressions in the three datasets only indicate one referent, so the trained model cannot ground natural language instructions with multiple target objects.

Considering the richness and diversity of natural language, and the relatively simple expressions in the three datasets, the trained referring expression comprehension model can not achieve complex natural language grounding. To this end, we combine scene graph with the referring expression comprehension network to ground unconstrained and sophisticated natural language.

Scene graph was introduced in Johnson et al. (2015), in which the scene graph is used to describe the contents of a scene. Compared with dependency parsing, scene graph parsing generates less linguistic constituents. Given a natural language sentence, scene graph parsing aims to parse the natural language sentence into scene graph legends, which consist of nodes comprise objects with attributes and edges express the relations between target and objects. For instance, for the sentence “red apple next to the bottle,” the generated scene graph legend contains node (“red apple”) and node (“bottle”), and edge (“next to”).

Formally, a scene graph legend is defined as a tuple G(S) = (N(S), E(S)), where N(S) = {*N*_1_(*S*), *N*_2_(*S*), ., *N*_*n*_(*S*)} is a set of nodes that encode objects with attributes, and E(S) = {*E*_1_(*S*), *E*_2_(*S*), ., *E*_*m*_(*S*)} is a set of edges that express the relations between objects. Specifically, a node *N*_*i*_(*S*) ⊆ *n*_*i*_ × $A_{i}$ represents attribute $A_{i}$ of an object *n*_*i*_ (e.g., red apple). An edge *E*_*i*_(*S*) ⊆ (*n*_*o*_ × *R* × *n*_*s*_) denotes the relation *R* between a subject *n*_*o*_ and an object *n*_*s*_, (e.g., next to).

In general, a scene graph parser can be constructed on a corpus consisting of paired node-edge labels. However, no such dataset is released for interactive natural language grounding. In order to ensure the natural language is parsed correctly, we adopt a simple yet reliable rule, i.e., word-by-word match, to achieve scene graph alignment. Specifically, for a generated scene graph, we check the syntactic categories of each word in a node and an edge by part of speech. A parsed node should consist of a noun or an adjective, and an edge contains an adjective or an adverb. In practice, we adopt the language scene graph (Schuster et al., 2015) and the natural language toolkit (Perkins, 2010) to complete scene graph generation and alignment.

## 6. Experiments and Results

### 6.1. Referring Expression Comprehension Benchmark

#### 6.1.1. Datasets

We train and validate the referring expression comprehension network on RefCOCO, RefCOCO+, and RefCOCOg. The images of the three datasets were collected from MSCOCO dataset (Lin et al., 2014).

**RefCOCO** comprises 142,210 expressions for 50,000 referents in 19,994 images. We adopt the same split with (Yu et al., 2016). The dataset is divided into training, validation, and test, respectively. The training set contains 120,624 expressions for 42,404 objects in 16,994 images, the validation set has 10,834 expressions for 3,811 objects in 1,500 images. The testing partition comprises two splits, testA and testB. TestA includes 5,657 expressions for 1,975 objects in 750 person-centric images, while testB owns 5,095 object-centric expressions for 1,810 objects in 750 images.

**RefCOCO+** consists 141,564 expressions for 49,856 referents in 19,992 images. The split we use is same as (Yu et al., 2016). The training set consists of 120,191 expressions for 42,278 objects in 16,992 images, the validation partition contains 10,758 expressions for 3,805 objects in 1,500 images. TestA comprises 5,726 expressions for 1,975 objects in 750 images, and testB encompasses 4,889 expression for 1,798 objects in 750 images. Compared to RefCOCO, RefCOCO+ discards absolute location words and attaches more importance to appearance differentiators.

**RefCOCOg** contains 95,010 expressions for 49,822 referents in 25,799 images. As they are collected in a non-interactive pattern, the length of referring expressions in RefCOCOg are longer than RefCOCO and RefCOCO+. RefCOCOg has two types of data splitting, (Mao et al., 2016) splits the dataset into train and validation, and no test set is published. Another data partition (Nagaraja et al., 2016) split the dataset as training, validation, and test sets. We run experiments on the second division, in which the training set contains 80,512 expressions for 42,226 objects in 21,899 images, the validation split includes 4,896 expressions for 2,573 objects in 1,300 images, and the test partition has 9,602 expressions for 5,023 objects in 2,600 images.

#### 6.1.2. Experimental Setup

In practice, we set the length of the sentences to 10 for the expressions in RefCOCO and RefCOCO+, and pad with “pad” symbol to the expressions whose length is smaller than 10. We set the length of the sentences to 20 and adopt the same manner to process the expressions in RefCOCOg.

We employ “bert-large-uncased” model^1^ to generate contextualized word embedding *E*_*r*_. According to Devlin et al. (2019), the word embedding from the sum of the last four layers acquire better results than the embedding extracted from the last layer. We select the embedding of the sum of the last four layers of BERT as *E*_*r*_. Therefore, the obtained expression representation *q* ∈ ℝ^10 × 1024^ for RefCOCO and RefCOCO+, and *q* ∈ ℝ^20 × 1024^ for RefCOCOg.

Given an image and referring expression pair, we utilize the final ground score defined in Equation 12 to compute the matching score for each object in the image, and pick the one with the highest matching score as the correct one. We calculate IoU (Intersection over Unit) between the selected region and the ground truth bounding box, and select the IoU value larger than 0.5 as the correct visual grounding.

We train our model with Adam optimizer with β_1_ = 0.9 and β_2_ = 0.999, we set the initial learning rate 0.0004 and decay every 5,000 iterations with weight decay 0.0001, and the total number of iterations is up to 30,000.

#### 6.1.3. Ablation Analysis

We adopt different combinations to validate the performance of each module, the results are shown in Table 1. According to (Yu et al., 2018b) and (Yu et al., 2018a), the models trained by the deep features extracted from VGG16 (Simonyan and Zisserman, 2014) generates lower accuracy than the features generated by ResNet101, so we do not train our model use VGG features.

**Table 1:** Ablation studies of our model using different module combinations.

		**RefCOCO**			**RefCOCO+**			**RefCOCOg**	
		**val(%)**	**testA(%)**	**testB(%)**	**val(%)**	**testA(%)**	**testB(%)**	**val(%)**	**test(%)**
1	sub(ProjFeat)+loc	79.28	79.57	80.37	64.77	65.29	62.41	69.63	69.28
2	sub(ProjFeat)+loc+rel	79.99	80.24	80.82	64.89	66.00	63.57	70.14	69.96
3	sub(SemanAware)+loc	80.59	80.61	81.73	64.20	65.89	63.47	72.94	72.72
4	sub(SemanAware)+loc+rel	81.24	81.42	82.20	65.11	66.03	63.76	72.98	72.76
5	sub(ProjFeat)+loc+rel+LangAtten	81.83	82.10	82.20	66.42	67.46	63.84	73.33	72.81
6	sub(SemanAware)+loc+rel+LangAtten	**83.51**	**83.74**	**83.18**	**68.16**	69.66	**64.66**	**76.00**	**74.81**
7	sub(SemanAware)+loc+rel+LangAtten(I)	83.25	82.55	82.55	67.77	**69.70**	64.00	74.53	73.61

*The bold values show the best grounding accuracy on each dataset split acquired by the proposed network*.

First, we validate the performance of our model from the visual perspective. We concatenate the project feature *f_v_^′^* and location representation *u*_*loc*_ as the visual representation for each region, and adopt the output of the BiLSTM as the representation for expressions. We set this combination as the baseline, and the results are listed in Line 1. We then add relation representation *u*_*rel*_ to evaluate the benefits of the relation module, and the results are listed in Line 2.

Second, we test the effectiveness of the visual semantic-aware network. We adopt the semantic-aware visual representation $f_{v}^{S}$ combined with the location and relation representation, respectively. Compared to Line 1 and Line 2, the results listed in Line 3 and Line 4 show the benefits of the visual semantic-aware network, and the accuracies are improved by nearly 2%.

Third, We employ two manners to evaluate the performance of the language attention network. We first select *f_v_^′^* as the visual representation for the target candidate, and combine the language attention network with the target localization module. It is clear that the results outperform than the results listed in Line 2. An interesting finding is that the results listed in Line 4 are close to Line 5, which also demonstrates the benefits of the visual semantic-aware network. We then adopt $f_{v}^{S}$ to represent the target candidate, and coalesce the language attention network with the other two modules. This combination acquires the best accuracies on the three datasets.

Fourth, we compare the influence of different word embeddings. We extract the embeddings from the last layer of BERT as the contextual representation for expressions and feed them into the language attention network, we denote this word embedding as LangAtten(I). Line 7 illustrates the obtained results. Compared with Line 6, the results show the advantage of the embeddings generated from the sum of the last four layers of BERT.

**Figure 3:**
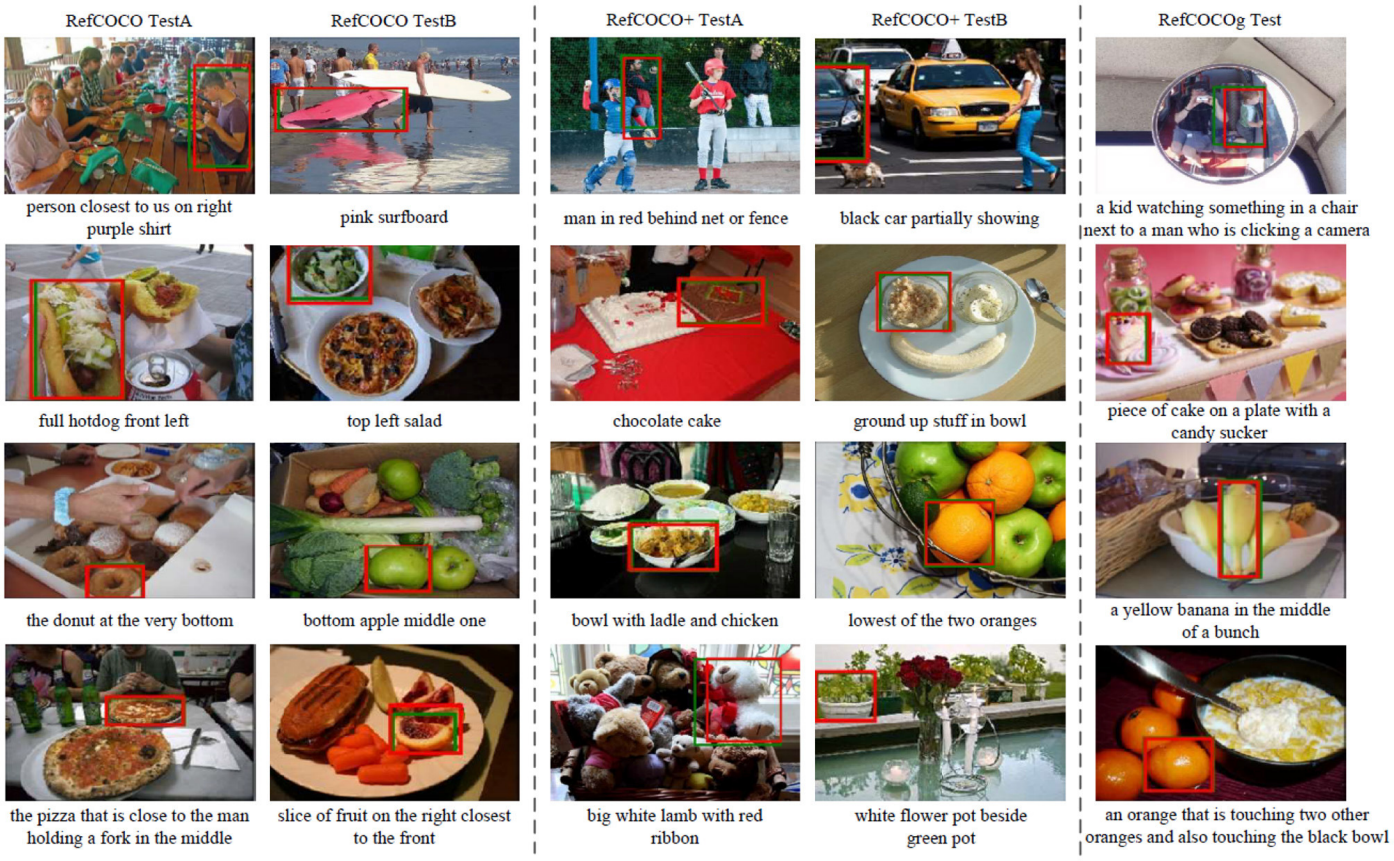
Example results of referring expression comprehension on test sets of RefCOCO, RefCOCO+, and RefCOCOg. Referring expressions are listed under the related images. In each image, the red box represents the correct grounding, and the green bounding box denotes the ground truth.

Finally, we list some example results acquired by the referring expression comprehension network in Figure 3. According to the experimental results, the presented model is able to locate the target objects for complex referring expressions, as shown in the experiments on RefCOCOg. As shown in Table 1, compared with the results on RefCOCO+ and RefCOCOg, our model acquires better results on RefCOCO. We found the expressions in RefCOCO frequently utilize the attributes and location information to describe objects, while the expressions in RefCOCO+ abandon the location descriptions while utilize more appearance difference to depict objects. In addition, the expressions in RefCOCOg involve the descriptions of neighborhood objects of referents and frequently use the relation between objects to define the target objects.

#### 6.1.4. Comparison With State-of-the-Art

Table 2 lists the results acquired by the proposed model and the state-of-the-art models. The table is split into two parts over the rows: the first part lists the approaches without introducing the attention mechanism. The second illustrates the results acquired by attention integrated models.

**Table 2:** Comparison with the state-of-the-art approaches.

		**RefCOCO**			**RefCOCO+**			**RefCOCOg**		
		**val(%)**	**testA(%)**	**testB(%)**	**val(%)**	**testA(%)**	**testB(%)**	val*(%)	**val(%)**	**test(%)**
1	visdif (Yu et al., 2016)	-	67.57	71.19	-	52.44	47.51	59.25	-	-
2	MMI (Mao et al., 2016)	-	63.15	64.21	-	48.73	42.13	55.16	-	-
3	attr+MMI+visdif (Liu et al., 2017)	-	78.85	78.07	-	61.47	57.22	69.83	-	-
4	Speaker (Yu et al., 2017)	79.56	78.95	80.22	62.26	64.60	59.62	72.63	71.65	71.92
5	Listener (Yu et al., 2017)	78.36	77.97	79.86	61.33	63.10	58.19	72.02	71.32	71.72
6	VC (Zhang et al., 2018)	-	78.98	82.36	-	62.56	62.90	73.98	-	-
7	DDPN+VGG16 (Yu et al., 2018b)	76.9	67.5	73.4	67.0	50.2	60.1	-	-	-
8	DDPN+ResNet101 (Yu et al., 2018b)	80.1	72.4	76.8	70.5	54.1	64.8	-	-	-
9	CMN (Hu et al., 2017)	-	-	-	-	-	-	69.30	-	-
10	AccuAtten (Deng et al., 2018)	81.27	81.17	80.01	65.56	68.76	60.63	73.18	-	-
11	PLAN (Zhuang et al., 2018)	81.67	80.81	81.32	64.18	66.31	61.46	69.47	-	-
12	MAttNet+VGG16 (Yu et al., 2018a)	80.94	79.99	82.30	63.07	65.04	61.77	73.08	73.04	72.7
13	LGRANs (Wang et al., 2019)	82.0	81.2	**84.0**	66.6	67.6	**65.5**	-	75.4	74.7
14	VisSemanAware+LanAtten	**83.51**	**83.74**	83.18	**68.16**	**69.96**	64.66	-	**76.00**	**74.81**

*The bold values show the best grounding accuracy on each dataset split*.

First, the proposed model outperforms the other approaches and acquire competitive results with the current state-of-the-art approach (Wang et al., 2019). (Wang et al., 2019) built the relationships between objects via a directed graph constructed over the detected objects within images. Based on the directed graph, this work identified the relevant target candidates by a node attention component and addressed the object relationships embedded in referring expressions via an edge attention module. This work focused on exploiting the rich linguistic compositions in referring expressions, while neglected the semantics embedded in visual images. In our proposed network, we address both the linguistic context in referring expressions and visual semantic in images.

Second, through the experiments on the three datasets, the introduced model acquires better results on RefCOCO compared with the results on RefCOCO+ and RefCOCOg. The expressions in RefCOCO frequently utilize the location or other details to describe target objects, the expressions in RefCOCO+ abandon the location descriptions and adopt more appearance difference. While the expressions in RefCOCOg attach more importance to the relation between the target candidates and their neighborhood objects to depict the target objects.

**Figure 4:**
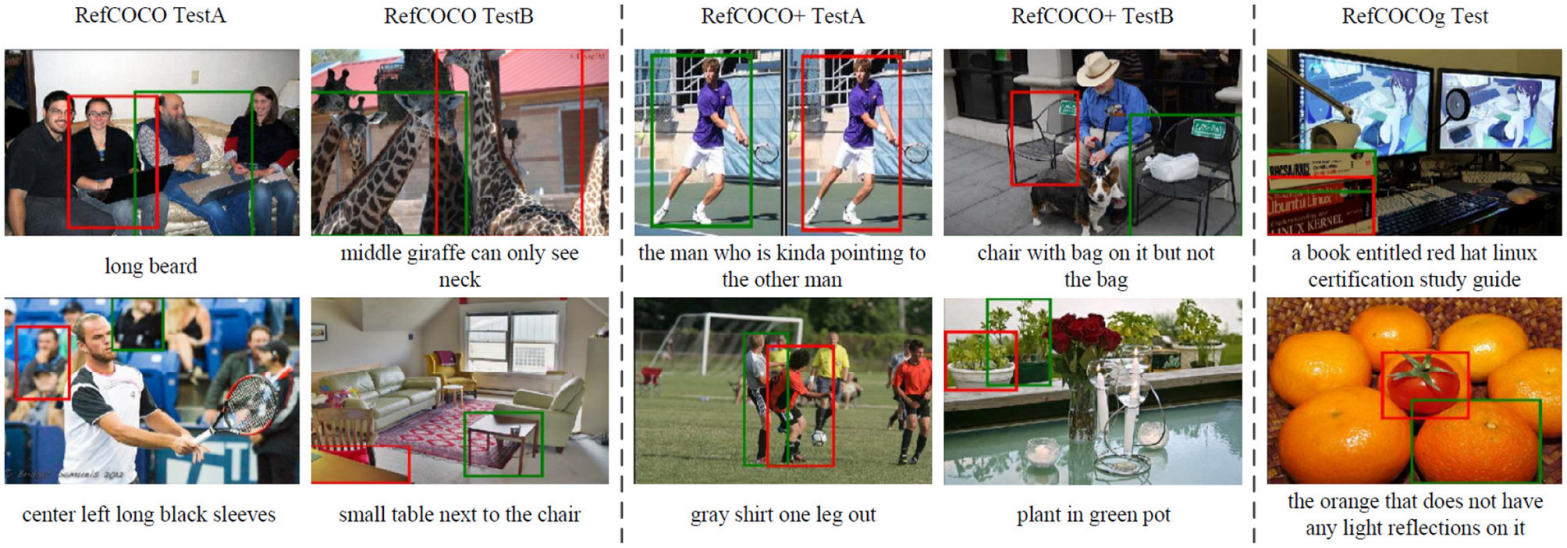
Examples of incorrect predictions. The red boxes show wrong visual groundings, and the green boxes denote the ground truth bounding boxes.

Finally, we show some failure cases on the three datasets in Figure 4. For complex expression, similar to “small table next to the chair,” our model generates closest weights for “table” and “chair.” Moreover, to locate the object with vague visual features, such as the target for “black sleeves” in the first left image and “guy leg out” in the third image of the second row, our model frequently generates wrong predictions. For the long expression and image with the complex background, such as the two images in RefCOCOg, our model fails to generate correct predictions.

### 6.2. Interactive Natural Language Grounding

We evaluate the effectiveness of the presented interactive natural language grounding architecture in two different manners. First, we collect 133 indoor scenarios from the test datasets of RefCOCO, RefCOCO+, and RefCOCOg, and collect 187 expressions that contain 2 referents for the selected images. These collected scenarios consist of the household objects that can be manipulated by robots. The average length of the expressions for MSCOCO images is 10.75. Second, we use a Kinect V2 camera to collect 30 images which are composed of the commonly used household objects and can be manipulated by robots. We collect 228 expressions, which contain 132 expressions with 2 referents and 96 expressions with 3 targets. The average number of words in these expressions is 14.31.

In order to collect diverse expressions for the collected images, we recruit 10 participants and show them different scenarios. For the MSCOCO images, we ask the participants to give expressions to depict two specific targets for each scenario, such as “the bottom row second donut from the left and the bottom rightmost mug.” For the self-collected scenarios, we ask the participants to give expressions with two or three referents for each image, for example, “move the red apple outside the box into the box and take the second water bottle from the right.” The collected working scenarios and expressions can be downloaded from the following link: https://drive.google.com/open?id=1k4WgpHTGaYsIE9mMmDgE_kiloWnYSPAr.

**Figure 5:**
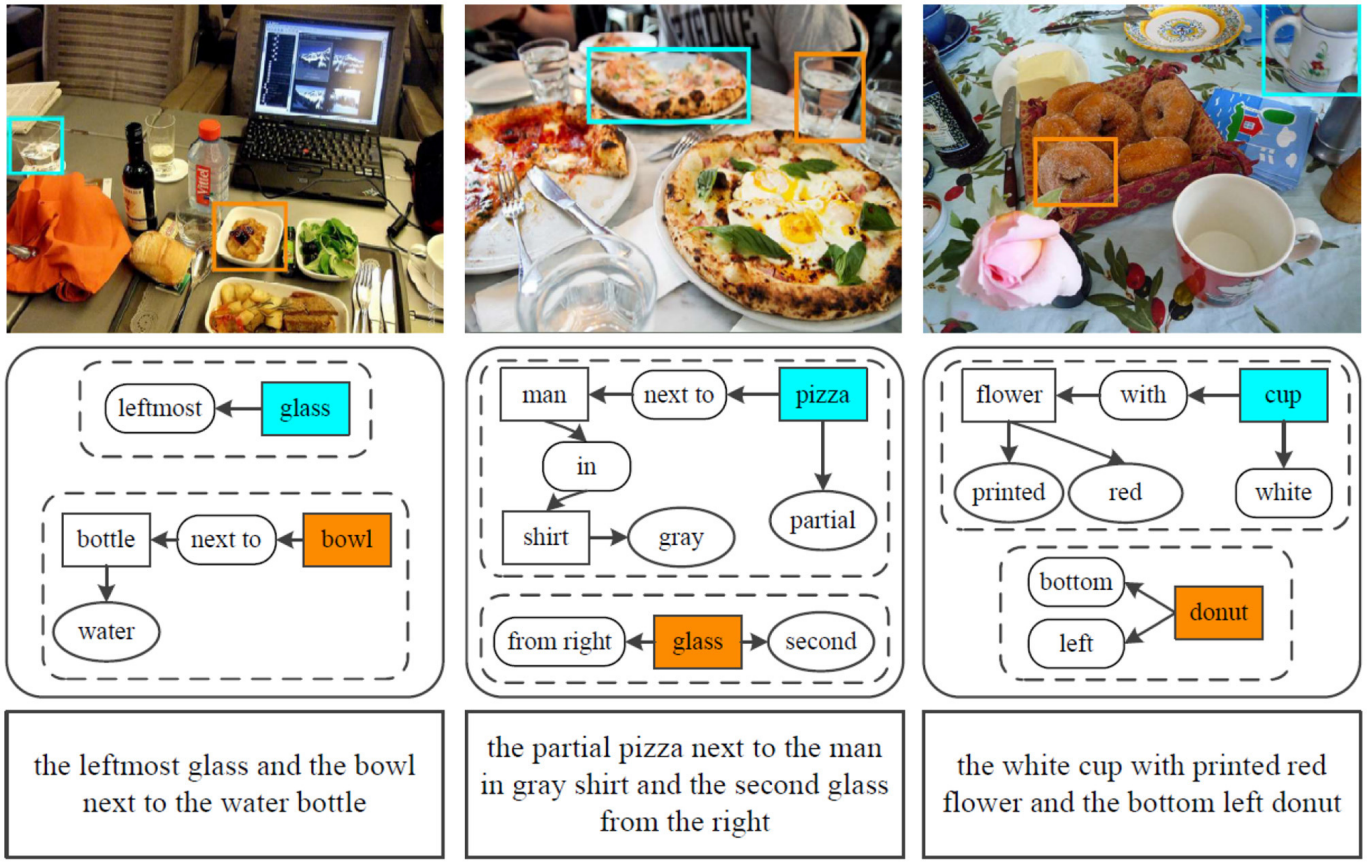
Example results of interactive natural language grounding on MSCOCO images. The input natural language instructions are listed in the third row with rectangle, the scene graph parsing results are shown in the second row with rounded rectangle.

In order to validate the performance of the proposed interactive natural language grounding architecture, we conduct grounding experiments on the collected indoor scenarios and natural language queries. We adopt the available scene graph parser source^2^ introduced (Schuster et al., 2015) to parse the complicated queries into scene graph legends (e.g., the parsing results listed in the rounded rectangles in the second row in Figure 5), and the trained referring expression comprehension model to locate target objects within given scenarios.

**Figure 6:**
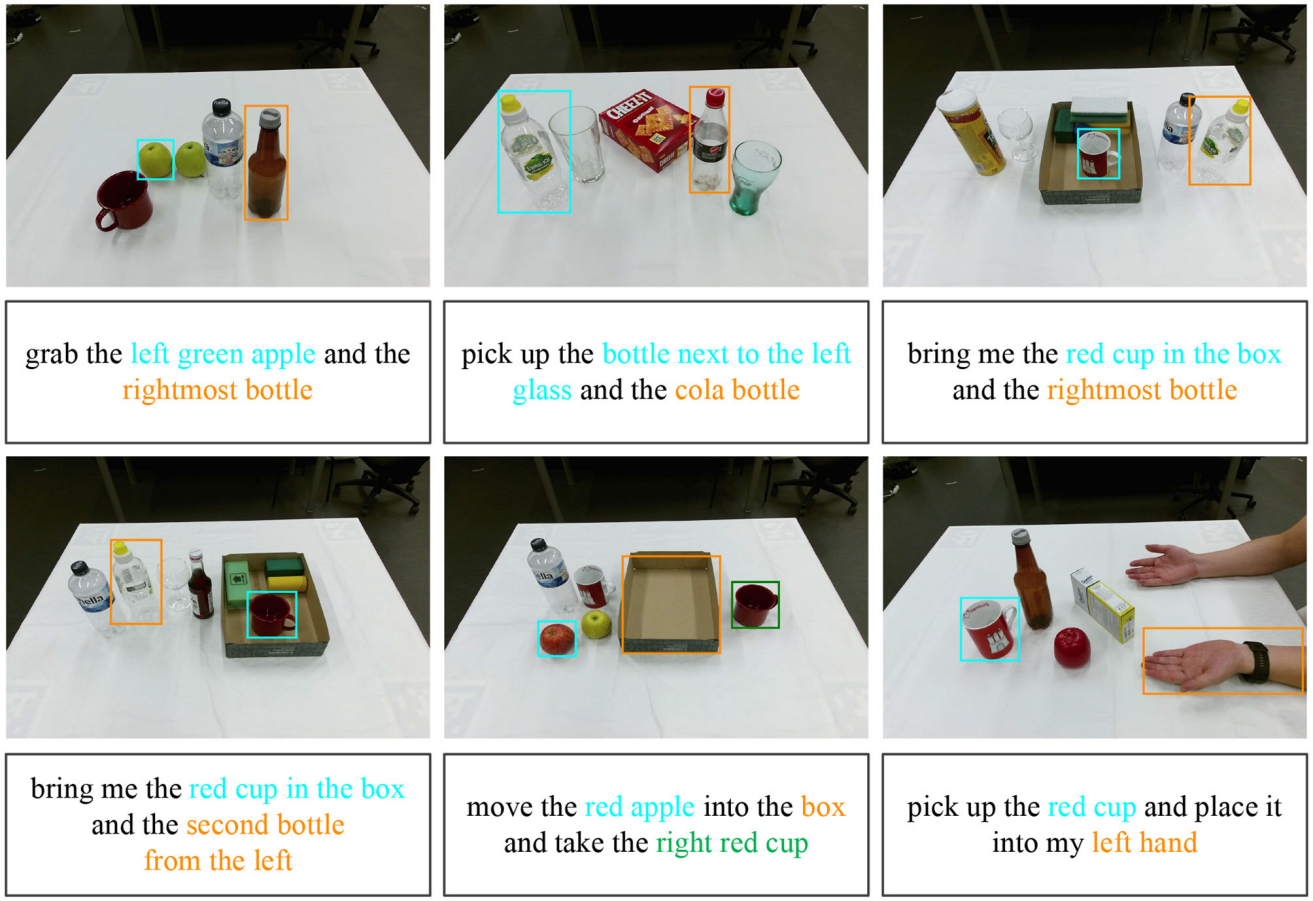
Example results of interactive natural language grounding on self-collected scenarios. The input natural language are listed in the rectangles, and the parsed scene graph legends are covered with related colors.

Figure 5 lists some grounding results on the collected MSCOCO images. We adopt the referring expression comprehension network trained on the three datasets to ground the collected expressions, respectively. The accuracies of the collected expressions grounding for MSCOCO images acquired by the three models are RefCOCO 86.63%, RefCOCO+ 79.41%, and RefCOCOg 80.48%. Figure 6 shows the grounding example results on the self-collected scenarios. The grounding accuracies attained by the three models are RefCOCO 91.63%, RefCOCO+ 87.45%, and RefCOCOg 88.44%. From these experimental grounding results, it is clear that the trained referring expression comprehension models have superior robustness.

Because of the properties of referring expressions in the RefCOCO, RefCOCO+, and RefCOCOg, the model trained on RefCOCO acquired the best results on the self-collected working scenarios. Instead of discarding spatial location words in expressions provided by RefCOCO+ expressions, and highlighting relationships between objects in RefCOCOg expressions, the collected expressions are more similar to the expressions in RefCOCO. Specifically, we take into consideration of descriptions of target attributes, spatial location of targets within images, and the relation between targets and their neighborhood objects in the collected natural language queries.

We also analyze the failure target object grounded working scenarios and related expressions, we found that the expressions with more “and” cannot be parsed correctly. For instance, the expression “take the apple between the bottle and the glass and the red cup” will be parsed into four nodes “apple,” “bottle,” “glass,” and “red apple,” while the relation between “apple,” “bottle,” and “glass” is lost, which leads to a failure grounding.

## 7. Conclusion

We proposed an interactive natural language grounding architecture to ground unrestricted and complicated natural language queries. Unlike the existing methods for interactive natural language grounding, our approach achieved natural language grounding and queries disambiguation without the support from auxiliary information. Specifically, we first presented a semantic-aware network for referring expression comprehension which is trained on three commonly used datasets in referring expressions. Considering the rich semantics in images and natural referring expressions, we addressed both visual semantic and textual contexts in the presented referring expression comprehension network. Moreover, we conducted multiple experiments on the three datasets to evaluate the performance of the proposed referring expression comprehension network.

Furthermore, we integrated the referring expression comprehension network with scene graph parsing to ground complicated natural language queries. Specifically, we first parsed the complicated queries into scene graph legends, and then we fed the parsed scene graph legends into the trained referring expression comprehension network to achieve target objects grounding. We validated the performance of the presented interactive natural language grounding architecture by implementing extensive experiments on self-collected indoor working scenarios and natural language queries.

Compared to the existing work for interactive natural language grounding, the proposed architecture is akin to an end-to-end approach to ground complicated natural language queries, instead of drawing support from auxiliary information. And the proposed architecture does not entail time cost as the dialogue-based disambiguation approaches. Afterward, we will improve the performance of the introduced referring expression comprehension network by exploiting the rich linguistic compositions in natural referring expressions and exploring more semantics from visual images. Moreover, the scene graph parsing module performs poorly when parsing complex natural language queries, such as sentences with more “and,” we will focus on improve the performance of the scene graph parsing. Additionally, we will exploit more effective methods to ground more complicated natural language queries and conduct target manipulation experiments on a robotic platform.

## Data Availability Statement

The datasets generated for this study are available on request to the corresponding author.

## Author Contributions

JM designed the study, wrote the initial draft of the manuscript, trained the referring expression comprehension network, completed the scene graph parsing module, implemented the referring expression comprehension experiments, and designed interactive natural language architecture validation experiments. JL, ST, and QL provided critical revise advices for the manuscript. All authors contributed to the final paper revision.

## Conflict of Interest

The authors declare that the research was conducted in the absence of any commercial or financial relationships that could be construed as a potential conflict of interest.

## Footnotes

^1^https://github.com/huggingface/pytorch-pretrained-BERT

^2^https://nlp.stanford.edu/software/scenegraph-parser.shtml
